# Herons in Aquafarms Are More Fearful of Humans

**DOI:** 10.1002/ece3.70924

**Published:** 2025-01-29

**Authors:** Shuang Yang, Sidan Lin, Wei Liang

**Affiliations:** ^1^ Ministry of Education Key Laboratory for Ecology of Tropical Islands, Key Laboratory of Tropical Animal and Plant Ecology of Hainan Province, College of Life Sciences Hainan Normal University Haikou China; ^2^ Key Laboratory for Conserving Wildlife With Small Populations in Yunnan, College of Forestry Southwest Forestry University Kunming China

**Keywords:** aquafarm, Ardeidae, flight initiation distance, human–wildlife conflict

## Abstract

Herons (Ardeidae), an important group in wetland ecosystems, have a particularly strong relationship with humans. However, their predation on farmed fish and shrimp in aquafarms can lead to economic losses for local fish farmers. Consequently, notable conflicts arise between fish farmers and herons. Fish farmers adopt various measures to deter herons from “stealing” their fish; however, there is limited research on the behavioral responses of herons under such pressures of human disturbance. Flight initiation distance (FID) is an important manifestation of avian anti‐predation behavior and serves as an indicator of birds' tolerance to human disturbance. This study investigated the potential variations in tolerance to human disturbance between the little egret ( *Egretta garzetta* ) and the Chinese pond heron ( *Ardeola bacchus* ) in “stealing” environments (aquafarm environments) and non‐aquafarm environments in Hainan and Guangdong Provinces, south China, using FID as an indicator. Herons in aquafarm environments were less tolerant (having longer FIDs) to human disturbance compared to herons in non‐aquafarm environments. The main cause of this phenomenon could be the harsh deterrents used by humans to prevent herons from “stealing” fish. These deterrents increase the predation risk for herons when “stealing” in aquafarms, causing them to exhibit less tolerance to human disturbance. Our study increases understanding of the behavioral response patterns of herons in human–wildlife conflicts and provides valuable insights for more scientific and rational management and protection of Ardeidae species.

## Graphical abstract

Fish farmers adopt various measures to deter herons from “stealing” their fish; however, there is limited research on the behavioral responses of herons under such pressures of human disturbance.Herons in aquafarm environments were less tolerant (having longer flight initiation distance) to human disturbance compared to herons in non‐aquafarm environments.Our study contributes to understanding of the behavioral response patterns of herons in human–wildlife conflicts and provide valuable insights for rational management and protection of Ardeidae species.

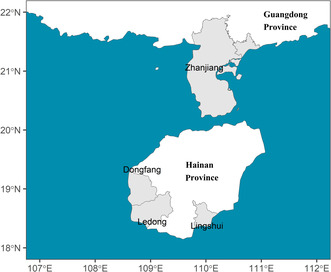

## 1 Introduction

In conjunction with urbanization and increasing human activities, human–wildlife conflict has detrimental effects on both humans and animals and is considered as a serious threat to numerous wildlife species in the present day (Dickman 2010). Animals adapt their behavior in response to human disturbance in their environment (Frid and Dill 2002). Behavioral plasticity increases the adaptability of wildlife living in areas of human disturbance (Barrett, Stanton, and Benson‐Amram 2018). For example, urban wildlife that becomes accustomed to human disturbance through learning may exhibit increased boldness or tolerance over time (Lowry, Lill, and Wong 2013).

In avian research, flight initiation distance (FID) is commonly used as an indicator to evaluate birds' tolerance to human disturbance (Joanna and Michael 1991; Møller 2008a). FID is considered an important manifestation of anti‐predation behavior. It refers to the distance between the predator (human) and the target animal when the target animal escapes or flees as the predator approaches (Gotanda, Turgeon, and Kramer 2009; Hediger 1934; Møller et al. 2015). FID is also considered a behavioral reflection of life history trade‐offs (Møller 2014): to optimize their fitness, animals make trade‐offs between staying put and fleeing (Møller and Liang 2013). Predictably, animals with shorter FIDs are more susceptible to predation, whereas those with longer FIDs are at a lower risk of predation. However, this lower risk is offset by the disadvantage of experiencing frequent interruptions to their foraging activities or ongoing behaviors (Møller, Nielsen, and Garamzegi 2008). The bird demonstrates greater tolerance and adaptability to human disturbance as the FID decreases (Møller 2008b). Because FID can measure the risk‐taking ability of target animals in the face of predators, it has been widely used in a variety of animal groups to understand how to minimize human disturbance and establish buffer zones, and in other topics of ecological conservation (Burger 1981; Glover et al. 2011; Weston et al. 2012).

FID is specific to each species, and different bird species have different FIDs (Blumstein et al. 2005, 2003). In addition to interspecific differences, factors such as perch height (Fernández‐Juricic, Vaca, and Schroeder 2004), habitat characteristics (Fernández‐Juricic, Vaca, and Schroeder 2004; Samia et al. 2015; Samia, Pape Møller, and Blumstein 2015), morphological traits (Blumstein 2006; Fernández‐Juricic et al. 2006; Glover et al. 2011; Møller and Erritzøe 2010), flocking behavior (Morelli et al. 2019), life history traits (Møller and Garamszegi 2012), and predator‐related factors (Bateman and Fleming 2011; Geist et al. 2005) all influence the FID of some bird species. The process of urbanization can also affect the expression of FID in birds. For example, the FID of birds in urban areas is considerably shorter than that of birds in rural environments (Díaz et al. 2013; Møller 2008b; Møller et al. 2015). In the context of urbanization, human activities, and their impacts on the adaptability of birds to human disturbances, are receiving increasing attention. For instance, during the COVID‐19 pandemic, the widespread use of face masks led to birds demonstrating higher adaptability to individuals wearing masks, which suggest a novel aspect of short‐term adaptation of wildlife to human behavior, and that the learning ability of birds allow them to adjust their behaviors to adapt rapidly to the new environmental conditions induced by human behavior (Jiang et al. 2020; Yang et al. 2024).

Herons (Ardeidae) constitute a significant component of wetland ecosystems and exhibit a notably close relationship with humans (Liang, Wong, and Wong 2006). In field studies, we found that herons often forage in environments such as aquafarms and rice fields. In aquafarm environments, herons prey on fish, shrimp, and other aquatic animals, causing direct economic losses to local fish farmers (Werner, Harrel, and Wooten 2005). This results in notable conflicts between fish farmers and herons. To reduce such economic losses in aquaculture, fish farmers adopt a variety of measures to prevent herons from “stealing.” For example, in China, aquafarm personnel frequently employ various tactics to deter herons, such as creating loud noises using gongs and drums, shouting, playing music, or setting off firecrackers, as well as setting traps to capture the birds (https://baijiahao.baidu.com/s?id=1812055088017792116&wfr=spider&for=pc). In contrast, in rice fields and other non‐aquafarm environments, farmers do not object to the presence of herons. Unlike other birds that feed on grains, herons do not cause direct economic losses to the farmers. Thus, herons in non‐aquafarm environments are less likely to be actively driven away or captured by humans (Liang, Wong, and Wong 2006). Previous studies mainly focused on the foraging behavior and monetary impacts of herons and egrets at baitfish aquaculture facilities in in foreign regions (e.g., Taylor et al. 2010; Werner, Harrel, and Wooten 2005). The key research question in this study was whether herons exhibit lower tolerance to human disturbances due to stronger disturbance stimuli and greater survival threats in aquafarms nearby the coastal areas in Hainan and Guangdong Provinces, south China. This study focused on two common species of herons, little egrets ( *Egretta garzetta* ) and Chinese pond herons ( *Ardeola bacchus* ). By measuring FID in aquafarm and non‐aquafarm environments, we aimed to identify potential differences in the herons' tolerance to human disturbances in “stealing” environments (aquafarm environments) compared to other environments (non‐aquafarm environments). Given the evident differences in levels of human disturbances in aquafarm and non‐aquafarm environments, we predicted that herons in aquafarm environments are likely to exhibit longer FIDs and lower tolerance to human proximity.

## 2 Materials and Methods

### 2.1 Study Area and Study Species

**FIGURE 1:**
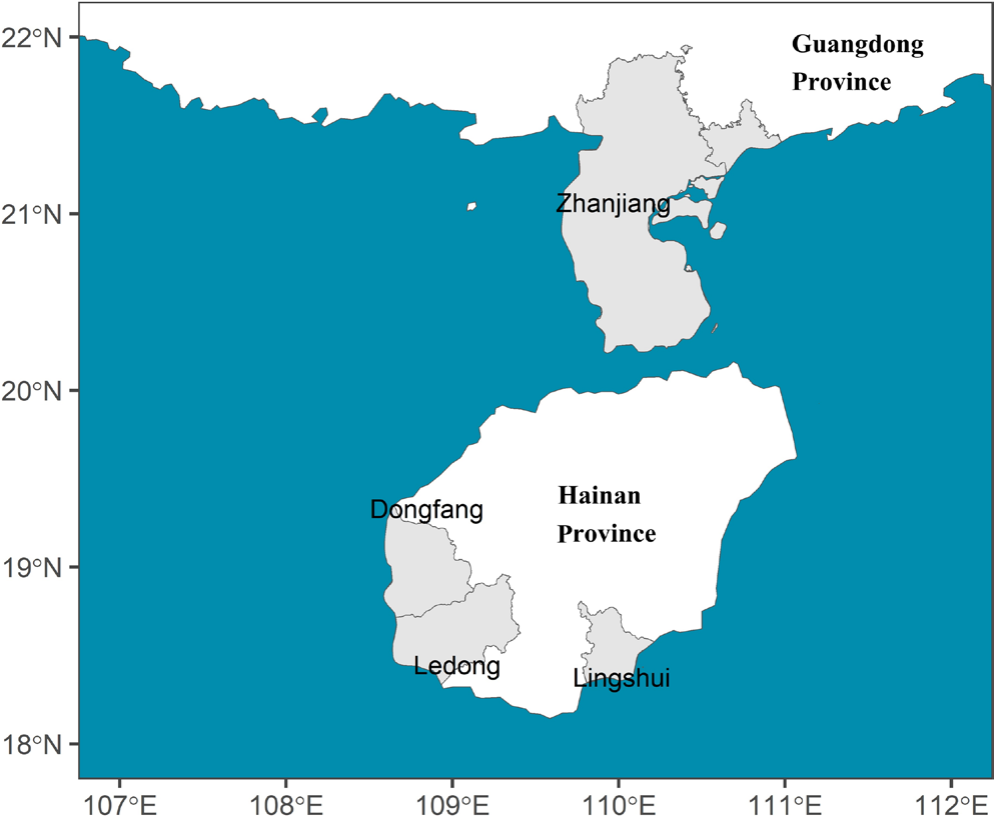
The location map of the study areas.

This study was conducted in the coastal areas of Lingshui and Ledong counties and Dongfang City in Hainan Province and Zhanjiang City in Guangdong Province, south China (Figure 1).

Hainan (108°37′–111°03′ E, 18°10′–20°10′ N) is an island province located in the southernmost part of China, bordered on the north by the Qiongzhou Strait and Guangdong Province and on the west by the Gulf of Tonkin, opposite Vietnam, with an area of 33,900 km^2^. Hainan Island has a tropical monsoon marine climate, characterized by indistinct seasons, moderate summer temperatures, mild winters, minimal annual temperature variation, and a high annual average temperature. The annual average temperature ranges from 22.5°C to 25.6°C, with an average annual rainfall of approximately 1640 mm (Liang, Wong, and Wong 2006).

Zhanjiang (109°40′–110°58′ E, 20°13′–21°57′ N) is located in the southwestern part of Guangdong Province, encompassing the entire Leizhou Peninsula and part of the northern peninsula. Zhanjiang is situated in the low‐latitude region and experiences a tropical monsoon climate, which is regulated by the marine climate throughout the year, with mild winters and moderate summers. The annual average temperature ranges from 22.7°C–23.5°C. Average annual rainfall is 1395.5–1723.1 mm (Tian et al. 2024).

Herons (family Ardeidae), belonging to the order Pelecaniformes, are medium‐ to large‐sized wading birds. In China, there are nine genera and 26 species of herons (Zheng 2023). They typically inhabit wetland environments such as rivers, lakes, mudflats, and marshes. The widespread distribution of herons renders them as optimal candidates for experimental research aimed at studying differences in anti‐predation behavior across various geographic environments. The body length of little egrets is 55–68 cm, and they often inhabit various wetlands such as lakes, swamps, and rice fields; the body length of Chinese pond herons is 42–52 cm, can adapt to various wetland environments and often lives alone or in small groups (Liu and Chen 2021). In this study, these two heron species are common in the southern region of China, were used as experimental subjects.

### 2.2 Field Data Collection

This study was conducted from December 2021 to February 2022. Surveys of bird FID were carried out under suitable and favorable weather conditions, between 7:00 a.m. and 18:00 p.m. (UTC/GMT +8.00). Habitat type was recorded as either aquafarms or non‐aquafarms (which included farmlands and other areas). During the experiment, the experimenter wore black clothing to avoid the influence of clothing color on the FID of the birds (Gould et al. 2004; Zhou and Liang 2020). The FID data were measured using a standardized method (Blumstein 2006; Weston et al. 2012). Before the experiment, the two observers received extensive training to familiarize themselves with the standardized FID measurement process. In the study area, observers first used binoculars (Swarovski, EL 10 × 42 SV) to search for experimental targets along designated routes. When they found a target individual (or flock) foraging or resting on the ground, they first identified the species of the target. Then, they directly approached the target individual at a normal walking speed (approximately 0.5 m/s) until the target animal flew away. At this point, the distance between the observer and the location where the target animal took flight was measured using a laser rangefinder [Sndway SW‐M500, Sndway Technology (Guangdong) Co. Ltd., Shenzhen, China] and recorded as the FID. The specified criteria for measuring FID was described by Zhou and Liang (2020): (1) The target birds needed to be in open spaces and away from surrounding shrubs and trees, to avoid any effect on FID from the birds' proximity to shelters; (2) The birds should be on the ground, or no higher than 2 m above the ground, and in a foraging or relaxed state; (3) If there was a flock consisting of multiple bird species, the target was excluded to avoid the effect of other species on the FID of the target individual; (4) When the experiment was conducted on a flock, the individual in the middle of the flock was selected for FID measurement; (5) The experimenter should ensure that the target individual was not influenced by other human activities, for example, approaching the target animal or making a lot of noise. An observer would continue walking in only one direction when experimenting with one certain habitat type, without sampling from the same position twice. This prevented repeat trials of the same individual in the same habitat.

### 2.3 Data Analysis and Statistics

To examine the effects of heron species, study area, habitat type (aquafarm vs. non‐aquafarm), and flock size on FID, the collected FID data were analyzed using a generalized linear mixed model (GLMM). The FID data were input as the target variable, with bird species, study area, habitat type, and flock size being set as fixed effects (see Yuan et al. 2024 for a similar approach). To account for variations attributable to different observers, the observer was included as a random effect. To further compare the FID between the two species of herons in different habitats, a one‐sample Kolmogorov–Smirnov test was used to check whether the data in each group followed a normal distribution. When the data followed a normal distribution, independent sample *t*‐tests were used for analysis. When the data did not follow a normal distribution, a non‐parametric Mann–Whitney *U*‐test was used to assess differences in FID between the groups. Spearman correlations between flock size and FID were calculated. Mean values are expressed as mean ± standard deviation (SD). The results were considered to be statistically significant at *p* < 0.05. All statistical tests were two‐tailed. Data analysis and graphs were performed using the software IBM SPSS 22.0 for Windows (IBM Corp., Armonk, NY, USA).

## 3 Results

We obtained a total of 561 FID data points for the two species of herons across different study areas and habitat types. Each group had at least 70 observations, meeting the requirements for data analysis. The sample size, minimum, median, maximum, mean, and standard deviation of FID for each group are shown in Table 1.

**TABLE 1:** Sample size (*N*=), minimum (Min), median (Med), maximum (Max), mean and standard deviation (SD) of flight initiation distance of two heron species in different study areas and habitat types.

Species	Study site	Habitat	*N*=	Min	Med	Max	Mean ± SD (m)
*Egretta garzetta*	Hainan	Aquafarm	72	39	65	130	71 ± 20
	Hainan	Farmland	117	19	43	76	43 ± 12
	Guangdong	Aquafarm	110	24	81	160	90 ± 25
*Ardeola bacchus*	Hainan	Farmland	118	4	23	58	24 ± 11
	Guangdong	Aquafarm	144	5	34	117	38 ± 22

**FIGURE 2:**
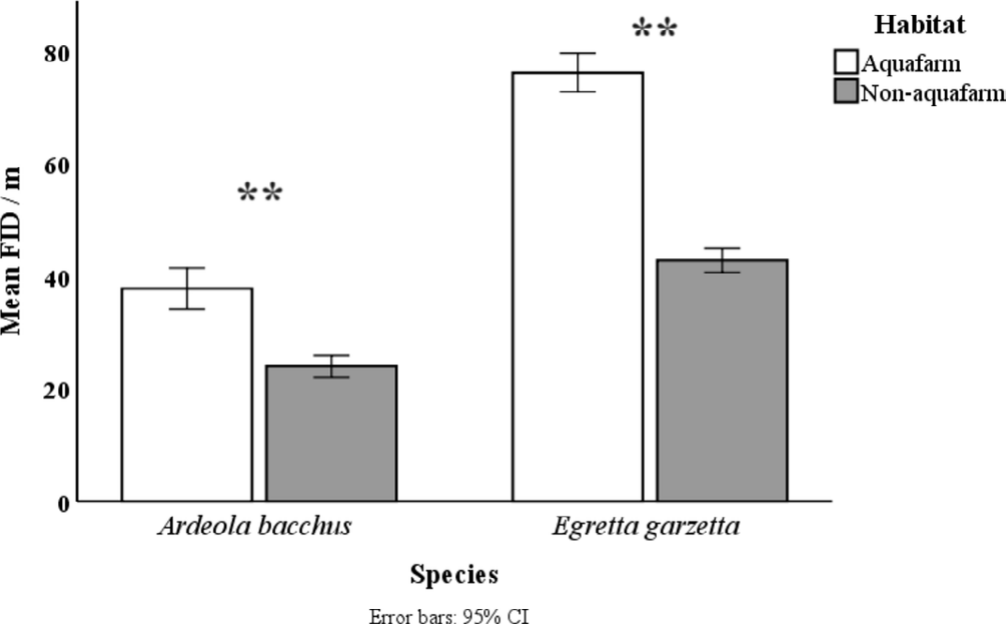
Post hoc comparison of flight initiation distance between two heron species in different habitats (** indicates a highly significant difference between the two groups, with *p* < 0.001).

When all data are combined for each species, the mean FID of the Chinese pond heron (31.59 ± 19.21 m; *n* = 262) differed significantly from that of the little egret (63.14 ± 25.60 m; *n* = 299; *p* < 0.001). Both little egrets and Chinese pond herons exhibited significantly longer FIDs in aquafarm environments compared to those in non‐aquafarm areas (Z_egrets_ = −11.993, *p* < 0.001; Z_pond heron_ = −5.348, *p* < 0.001, Mann–Whitney *U*‐test, Figure 2).

A linear mixed model analysis of the data containing all variables showed that two variables, “study area” and “flock,” did not have a significant effect on FID (P_Study area_ = 0.126; P_Flock size_ = 0.135). However, all the remaining factors had highly significant effects on heron FID (P_Species_ < 0.001, P_Habitat_ < 0.001) (Table 2). No significant Spearman correlation was found between the FID data of birds and their flock numbers (*r* = −0.026, *p* > 0.05).

**TABLE 2:** Generalized linear mixed model (GLMM) analysis on the effects of study area, habitat, species, and flock size on flight initiation distance of two heron species.

	***F***	**df1**	**df2**	***P***
Intercept	48.414	14	546	< 0.001
Species	313.280	1	546	< 0.001
Study area	2.348	1	546	0.126
Habitat	96.882	1	546	< 0.001
Flock size	1.479	11	546	0.135
	***t***	**df**	***P***	**95%CI**
Habitat				
Aquafarm versus farmland	9.843	546	< 0.001	[23.455–35.151]
species				
*Egretta garzetta* versus *Ardeola bacchus*	17.700	546	< 0.001	[27.178–33.963]

## 4 Discussion

In this study, we examined differences in tolerance to human approach between two species of herons in areas with varying intensities of human disturbance using aquafarms and non‐aquafarms as the study sites and FID as the observational indicator. Compared to the mild disturbance (such as farming) in non‐aquafarm environments, the high‐threat human disturbance (such as scaring and driving away) in aquafarm environments greatly reduced the herons' tolerance to human disturbance. This tolerance was not affected by bird flock size. These results fill a research gap in the study of anti‐predation behavior in herons and have important applications for the scientific management and conservation of heron species. For example, the residents around the protected area of herons should try to avoid setting off firecrackers and reducing activity noise. The boundary range of the protected area should be delineated according to the FID of herons.

Previous studies have indicated that different bird species exhibit variations in FID (Blumstein 2006). The significant difference in FID between the little egret and the Chinese pond heron in the present study further demonstrates the inter‐specific variability of FID. The apparent difference in FID between the two species of herons may be related to their body size, as there is generally a positive correlation between bird body size (weight) and FID; that is, the larger the body size, the longer the FID (Glover et al. 2011). Understandably, the taller herons can detect an approaching predator at a greater distance than shorter herons. Besides, larger birds are at greater risk of predation because they are more likely to be detected by predators. In addition, considering the fact that larger birds exhibit reduced agility compared to smaller birds (Witter, Cuthill, and Bonser 1994), it is possible that larger birds might be more inclined to flee sooner when encountering the same risk of predation (Blumstein 2006). Smaller birds have a higher energy requirement relative to their body size than larger birds, thus necessitating increased time spent in foraging activities (Bennett and Harvey 1987). To meet their dietary requirements, birds exhibit a willingness to take increased predation risks and enhance their foraging opportunities (Blumstein 2006). In the present study, the Chinese pond heron had a shorter FID. In addition to body size, another important factor was its camouflage during resting, which allowed it to effectively blend into the ground environment. In this study, the Chinese pond heron was better camouflaged than the little egret, except in a white sand environment. A previous study showed that bird species with camouflage have been reported to have shorter FIDs compared to their relatives without such traits (Møller, Liang, and Samia 2019). Camouflage helps animals hide from predators, providing a survival advantage to birds with camouflage over those with conspicuous colors (Troscianko et al. 2016; Møller, Liang, and Samia 2019). The protective coloration provides an additional benefit of allowing the prey to hide for a longer time and then escape unexpectedly from a stationary state, thus saving time and energy by reducing the frequency of responses to predator disturbance (Møller, Liang, and Samia 2019).

In the present study, both the little egret and the Chinese pond heron showed greater tolerance to human disturbance in non‐aquafarm environments. This could be attributed to the different intensities of human disturbance in these habitats. Aquafarms usually have large numbers of fish and shrimp, and the feed dispensed by farmers often leads to an abundance of floating feed particles on the water surface. Thus, aquafarms are essentially a food‐rich “buffet” for herons, typically attracting large numbers of herons and gulls. To deter these birds from “stealing” the farmed fish and feed, local fish farmers often use loud noises such as playing music, beating gongs and drums, and setting off firecrackers to scare and drive the birds away. Human disturbance is often perceived by animals as a predation risk (Frid and Dill 2002). The preventive measures on aquafarms apparently cause the herons to perceive humans as a severe threat, increasing the vigilance and sensitivity of the birds. This causes the birds to flee early to avoid the threat. In non‐aquafarm environments, such as farmland, human tolerance of herons is relatively high. The main targets of human scaring activities are small birds, such as sparrows ( *Passer montanus* ), that “steal” rice and other crops (Zhao et al. 2024). Owens, Stec, and O Hatnick (2012) suggested that chronic noise stimulation increases birds' level of perceived external threat (the “increased threat hypothesis”), and FID increases significantly when birds are exposed to noisy environments (Meillère, Brischoux, and Angelier 2015). It can thus be hypothesized that the scare tactics used by humans in aquafarm environments continually enhance the herons' perception of danger, leading to longer FIDs in these environments. The effect of learning on FID can also explain the observed difference in FID between the aquafarm and farmland environments. Weston et al. (2012) suggested that learning affects FID of birds in one of two ways. First, increased contact with humans leads to heightened sensitivity in birds, ultimately resulting in longer FIDs. This situation is typically associated with threatening and unpredictable stimuli. Conversely, the second effect is that increased contact with humans leads to greater adaptability to human activities, resulting in smaller FIDs. This situation is usually related to prolonged, mild, and predictable stimuli (Weston et al. 2012). In the present study, the aquafarm environment corresponds to the first scenario. The scare tactics and driving methods used by humans act as strong and threatening stimuli to the little egret, which increases the egrets' vigilance and sensitivity to human activities, resulting in larger FIDs. This situation is consistent with the behavioral responses of birds to hunting. Birds inhabiting protected areas exhibit higher tolerance to human approach compared to those in hunting areas (Magige et al. 2009). Thus, the scare tactics used in aquafarms for birds are similar to hunting, both being strongly threatening human disturbances. Non‐aquafarm environments, such as farmland, correspond to the second scenario. During the experiments, the researchers often observed large machinery plowing in the rice fields that attracted many birds (including herons) to forage for aquatic insects and other food brought to the surface by the machinery. Over time, this leads to increased adaptability of herons to human activities in farmland environments. Human activities, such as cultivation, in agricultural fields do not harm herons and sometimes provide additional food sources, so the gradual habituation and adaptation of herons to human activities in non‐aquafarm environments is ultimately manifested in a gradual decrease in FID.

Flock size, one of the important factors affecting the FID of birds, is well known to positively correlate with FID; FID increases with the number of individuals in the group (Laursen, Kahlert, and Frikke 2005; Scarton 2018; Mayer, Natusch, and Frank 2019; Morelli et al. 2019). It is reasonable that with the increase in the number of individuals in the group, the probability of detecting potential threats also increases (Stankowich and Blumstein 2005; Mayer, Natusch, and Frank 2019), enabling the group to evade threats earlier. Notably, sometimes the FID of a group is primarily influenced by the behavior of individuals within the group (Fernández‐Juricic, Jimenez, and Lucas 2002). Individuals who are more alert and agile within the group opting to flee from a potential threat may prompt similar behavioral reactions from those nearby, thereby affecting the entire group and leading to longer FIDs (Hingee and Magrath 2009; Weston et al. 2012). However, the “dilution effect hypothesis” suggests that the probability of a single individual being predated decreases with increasing group size (Roberts 1996; Sridhar, Beauchamp, and Shanker 2009). In addition, the “many eyes effect” suggests that individual vigilance decreases when birds can rely on the alertness of other group members (Roberts 1996). Many studies have indeed shown that FID shortens as group size increases (Jiang et al. 2020; Samia et al. 2015; Samia, Pape Møller, and Blumstein 2015). However, some studies have found no significant correlation between group size and FID (Guay et al. 2013; Kalb, Anger, and Randler 2019). In the present study, no significant association was found between group size and the FID of herons. Apart from the combined effects of the aforementioned hypotheses, another possible explanation for this phenomenon is that most of the observed experimental individuals were solitary (had a flock size of one: median = 1, mean ± SD = 1.77 ± 8.55 individuals), and larger group sizes were less frequently observed.

In summary, different intensities of human disturbance in aquafarm versus non‐aquafarm environments resulted in different levels of tolerance by bird species of the Ardeidae family. Herons in aquafarm environments were less tolerant to human disturbance compared to herons in non‐aquafarm environments. However, considering that this research exclusively focused on the Chinese pond heron and the little egret, further investigation is needed to determine whether the strongly threatening human disturbance in aquafarms also affects the anti‐predation behavior of other bird species, such as gulls, which often prey on fish in aquafarms and have conflicts with fish farmers. The preventive measures taken by fish farmers in this study require substantial human and material resources and affect the expression of related bird anti‐predation behaviors, which is not conducive to mitigating conflicts between birds and fish farmers. Therefore, it is imperative to prioritize the identification of efficient solutions that minimize financial losses for fish farmers, while also mitigating unnecessary human disturbance to herons. Implementing these measures would assist in mitigating human–wildlife conflicts and fostering a shift from antagonism to harmonious coexistence of urbanization and conservation efforts.

## Author Contributions

**Shuang Yang:** formal analysis (equal), investigation (equal), methodology (equal), writing – original draft (equal). **Sidan Lin:** data curation (equal), formal analysis (equal), investigation (equal), methodology (equal), writing – original draft (equal). **Wei Liang:** conceptualization (lead), supervision (lead), validation (equal), writing – review and editing (lead).

## Ethics Statement

The experiments comply with the current laws of China. No special permit was required for this study as it was not involved in animal or plant collection.

## Conflicts of Interest

The authors declare no conflicts of interest.

## Data Availability

Data and the code and model used for this study are provided as supporting material (Data Table S1) and can be found at https://figshare.com/s/2d4a21b0deaaf5a30c46 (doi: 10.6084/m9.figshare.27125412).
